# Association of C-type lectin 18 levels with extrahepatic manifestations in chronic HCV infection

**DOI:** 10.1038/s41598-018-35774-w

**Published:** 2018-11-23

**Authors:** Tsai-Ling Liao, Ya-Lang Huang, Yi-Ming Chen, Hsiu-Chin Lee, Der-Yuan Chen, Shie-Liang Hsieh

**Affiliations:** 1 Department of Medical Research, Taichung Veterans General Hospital, Taichung, Taiwan; 2 Ph.D. Program in Translational Medicine, National Chung Hsing University, Taichung, Taiwan; 3 Rong Hsing Research Center for Translational Medicine, National Chung Hsing University, Taichung, Taiwan; 4 Genomics Research Center, Academia Sinica, Taipei, Taiwan; 5 Department of Internal Medicine, Taichung Veterans General Hospital, Taichung, Taiwan; 6 Faculty of Medicine, National Yang Ming University, Taipei, Taiwan; 7 Translational Medicine Laboratory, Rheumatic Diseases Research Center, China Medical University Hospital, Taichung, Taiwan; 8 Rheumatology and Immunology Center, Department of Medicine, China Medical University Hospital, Taichung, Taiwan; 9 School of Medicine, China Medical University, Taichung, Taiwan

## Abstract

Mixed cryobulinemia (MC) is the most common chronic hepatitis C virus (HCV)-associated extrahepatic manifestation. C-type lectin 18 (CLEC18) is a novel secretory lectin that is abundantly expressed in hepatocytes and peripheral blood cells (PBCs). We investigated the associations between CLEC18 expression during HCV infection and the presence of extrahepatic manifestations. A total of 41 rheumatic patients with HCV infection (including 28 patients with MC syndrome), 45 rheumatic patients without infection, and 14 healthy subjects were enrolled. The CLEC18 levels in PBCs and serum were determined by using flow cytometry and enzyme-linked immunosorbent assay, respectively. Significantly higher CLEC18 levels were observed in patients with HCV infection (*P* < 0.001) and were positively correlated with HCV viral loads (γ = 0.56, *P* < 0.05). Among patients with HCV infection, significantly increased CLEC18 levels were observed in patients with MC syndrome, particularly in those with type II MC (*P* < 0.05). CLEC18 levels were associated with cryoglobulin and C4 levels (*P* < 0.05). CLEC18 was significantly associated with HCV infection, particularly in those with HCV-associated MC. CLEC18 levels were also positively correlated with MC disease activity, suggesting its involvement in MC pathogenesis. CLEC18 may be a novel indicator of HCV infection and a potential therapeutic target in rheumatic patients.

## Introduction

Hepatitis C virus (HCV) infection is a major health problem; the World Health Organization (WHO) estimates that at least 150–170 million people, approximately 3% of the global population, are chronically infected^1^. In Taiwan, the prevalence of HCV (1.8–5.5%) is higher compared with that in other Asian (e.g., Japan, Korea) or Western countries^2,3^. Most patients with HCV infection are unable to completely clear the HCV pathogen, which has resulted in a greater prevalence of chronic HCV infection^1^. In addition to liver damage, numerous HCV extrahepatic manifestations (HCV-EHMs) have been reported among patients with HCV infection^4,5^. A recent study demonstrated that the economic burden of HCV-EHMs has significantly increased, which may be partially abated by treatment using direct-acting antivirals^6^. Mixed cryoglobulinemia (MC), the most common extrahepatic manifestation of chronic HCV infection^7^, is characterized by the clonal proliferation of B cells and the formation of circulating immune complexes known, as cold-precipitable cryoglobulin^8^. Cryoglobulins has been detected in 30%–50% of patients chronically infected with HCV^8^, and HCV infection is observed in 70% to >90% of MC patients^9,10^.

Innate immunity plays a critical role in the response to infection and rheumatic diseases^11,12^. C-type lectins, which are carbohydrate-binding proteins, play an important role in the innate immune system by recognizing a wide range of pathogens^13^. Members of C-type lectins have been shown to be crucial pattern recognition receptors that recognize members of the flavivirus and influenza virus families. Spleen tyrosine kinase (Syk)–coupled C-type lectin member 5A (CLEC5A) is the pattern recognition receptor (PRR) for the dengue virus (DV)^14–16^, Japanese encephalitis virus (JEV)^17^, and influenza virus H5N1^18^. These viruses can activate CLEC5A to secrete abundant proinflammatory cytokines from macrophages and myeloid cells; blockade of CLEC5A can protect mice from DV- and JEV-induced lethality and neuroinflammation. Moreover, CLEC5A knockout mice exhibit increased resistance to H1N1-induced pulmonary inflammation and lethality compared to wild-type mice^18^. While the PRR for HCV remains unclear, the interaction of HCV with two members of the C-type lectin family (DC-SIGN and L-SIGN)^19,20^ contributes to the establishment or persistence of infection. We previously characterized CLEC18, a novel human C-type lectin^21^. CLEC18 belongs to group XV and is a soluble protein detectable in human serum and is upregulated during infection^21^. CLEC18 expression is abundant in normal hepatocytes but is absent in hepatocellular carcinoma (HCC)^21^. In addition, CLEC18 expression is abundant in human peripheral blood cells (PBCs). However, little is known regarding the association of CLEC18 expression with HCV infection or HCV-associated extrahepatic manifestations.

In this study, we investigated (1) the associations of CLEC18 expression with HCV infection; (2) the difference in CLEC18 levels in circulating PBCs among rheumatic patients with HCV infection, rheumatic patients without infection, and healthy control (HC); (3) the correlation of CLEC18 levels in PBCs and the presence of HCV-associated extrahepatic manifestations; and (4) the changes of CLEC18 levels in patients after a 6-month anti-rheumatic or anti-HCV therapy.

## Results

### Characteristics of the study cohort

A total of 100 participants, including 41 rheumatic patients with HCV infection, 45 rheumatic patients without infection, and 14 healthy subjects, were enrolled. Among 41 rheumatic patients with HCV infection, 26 (63.4%) had Sjögren’s syndrome (SS), 10 (24.4%) had rheumatoid arthritis (RA), and 5 (12.2%) had systemic lupus erythematosus (SLE). Among 45 rheumatic patients without infection, 20 (44.4%) had SS, 21 (46.7%) had RA, and 4 (8.9%) had SLE (Table 1).

**Table 1:** Demographic data and laboratory findings of rheumatic patients with chronic HCV infection, and without infection.

Variable	Chronic HCV Infection (n = 41)		Without infection
	With MC (n = 28)	Without MC (n = 13)	(n = 45)
Age at study entry (years)	64.2 ± 8.5	66.3 ± 5.0	65.8 ± 7.2
Gender (female, %)	21 (75.0)	10 (76.9)	33 (73.3)
Disease duration (years)	9.8 ± 5.3	10.2 ± 4.5	10.5 ± 3.7
Related rheumatic diseases			
Sjögren’s syndrome	21 (75.0)	5 (38.5)	20 (44.4)
Rheumatoid arthritis	5 (17.9)	5 (38.5)	21 (46.7)
Systemic lupus erythematosus	2 (7.1)	3 (23.1)	4 (8.9)
RF (IU/ml)	55.3 ± 28.9*	48.8 ± 21.2	45.6 ± 30.5
Anti-CCP antibody (positive, %)	11 (39.3)	5 (38.5)	21 (46.7)
ESR (mm/h)	28.7 ± 8.3	25.8 ± 3.9	23.3 ± 5.8
C4 (mg/dl)	16.3 ± 5.2*	35.2 ± 8.6	ND
ALT (U/l)	55.8 ± 32.8*	35.7 ± 15.6	ND
Anti-HCV antibody (positive, %)	28 (100.0)	13 (100.0)	0 (0)
HCV viral loads at baseline (106 IU/mL)	3.45 ± 3.48	3.84 ± 2.53	ND
Medications used at study entry			
corticosteroid	28 (100.0)	13 (100.0)	45 (100.0)
csDMARDs	28 (100.0)	13 (100.0)	45 (100.0)
TNF inhibitors	1 (3.6)	1 (7.7)	7 (15.6)
Rituximab	1 (3.6)	0 (0)	1 (2.2)
ombitasvir/paritaprevir/ritonavir	2 (7.1)	0 (0)	0 (0)

Values are mean ± SD or the number (%) of patients.

*P < 0.05 between chronic HCV infection with MC versus without MC or without infection.

ALT, alanine aminotransferase; C4, complement 4; CCP, cyclic citrullinated peptide; ESR, erythrocyte sedimentation rate; RF, rheumatoid factor; ND, nondetermined; csDMARDs, conventional synthetic disease-modifying anti-rheumatic drugs; TNF, tumor necrosis factor.

Among the 41 rheumatic patients with HCV infection, 28 patients (68.3%) had detectable serum MC; of these, 15 (53.6%) were type III and 13 (46.4%) were type II (Table 1). Increased levels of rheumatoid factor (RF) and alanine aminotransferase (ALT) values were measured in rheumatic patients with MC syndrome compared to those without MC (55.3 ± 28.9 vs. 48.8 ± 21.2 IU/ml, *P* < 0.05, and 55.8 ± 32.8 vs. 35.7 ± 15.6 U/l, *P* < 0.05, respectively). In contrast, significantly lower levels of complement component 4 (C4) were observed in rheumatic patients with MC compared to those without MC (16.3 ± 5.2 vs. 35.2 ± 8.6 mg/dl, *P* < 0.05). However, there were no significant differences in the age at entry, percentage of females, disease duration, positive rates of anti-cyclic citrullinated peptide (anti-CCP) antibodies or erythrocyte sedimentation rate (ESR) between rheumatic patients with and without HCV infection.

### Increased CLEC18 expression levels in hepatocytes with HCV infection

**Figure 1:**
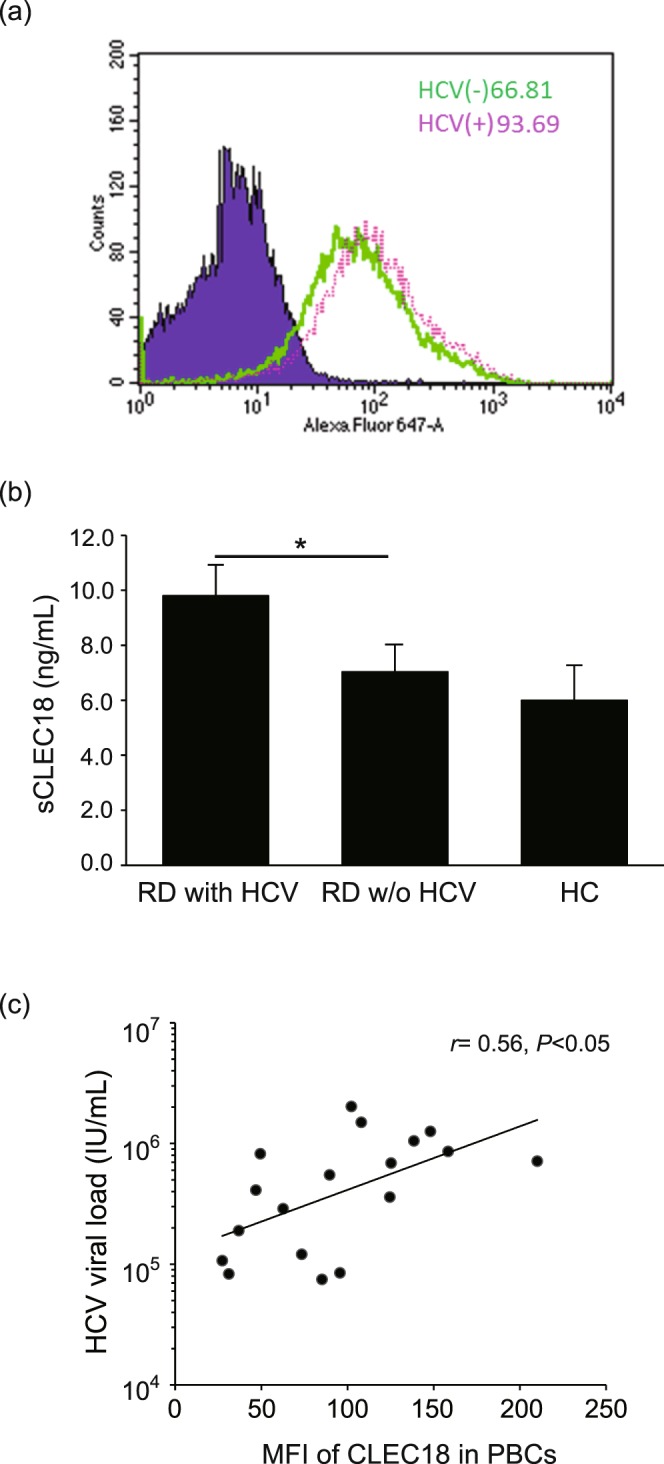
CLEC18 expression levels were associated with HCV infection. (**a**) Increased CLEC18 expression levels in hepatocytes with HCV infection. (**b**) Increased secretory CLEC18 levels in serum of patients with HCV infection. Data are presented as the mean ± SEM, rheumatic patients with HCV infection (n = 41) versus rheumatic patients without HCV infection (n = 45) or healthy control (n = 14), determined using the ANOVA test with the Scheffe correction. *P < 0.05. (**c**) CLEC18 expression levels in PBCs of patients with HCV infection are positively correlated with HCV viral loads. The correlation coefficient was calculated using Spearman’s correlation test.

To investigate whether CLEC18 expression is associated with HCV infection, Huh7.5 cells were infected with HCVcc JC1 strain at an MOI of 5. At 72 h postinfection, HCV-infected cells were collected for CLEC18 expression analysis by flow cytometry assay. Increased CLEC18 expression was detected in HCV-infected cells compared with uninfected cells (93.69% vs. 66.81%, Fig. 1a). Our results indicated increased CLEC18 expression levels in hepatocytes after infection with HCV.

### Increased CLEC18 levels in PBCs is positively correlated with HCV viral loads

To verify the association of CLEC18 and HCV infection, we measured serum CLEC18 levels in patients with or without HCV infection using ELISA. Higher levels of CLEC18 were observed in the serum of patients with HCV infection (9.8 ± 1.2 ng/ml, *P* < 0.05) compared to those without infection (7.0 ± 1.1 ng/ml) or healthy control subjects (6.0 ± 0.9 ng/ml) (Fig. 1b).

To further determine the association of CLEC18 expression in PBCs with HCV infection in humans, we examined the correlation between CLEC18 levels in PBCs and HCV viral loads in sera from rheumatic patients. Our results showed a positive correlation between CLEC18 expression levels and HCV viral loads in rheumatic patients (*r* = 0.56, *P* < 0.05, Fig. 1c).

### Increased CLEC18 expression levels in circulating T cells, monocytes, and neutrophils from rheumatic patients with HCV infection

**Figure 2:**
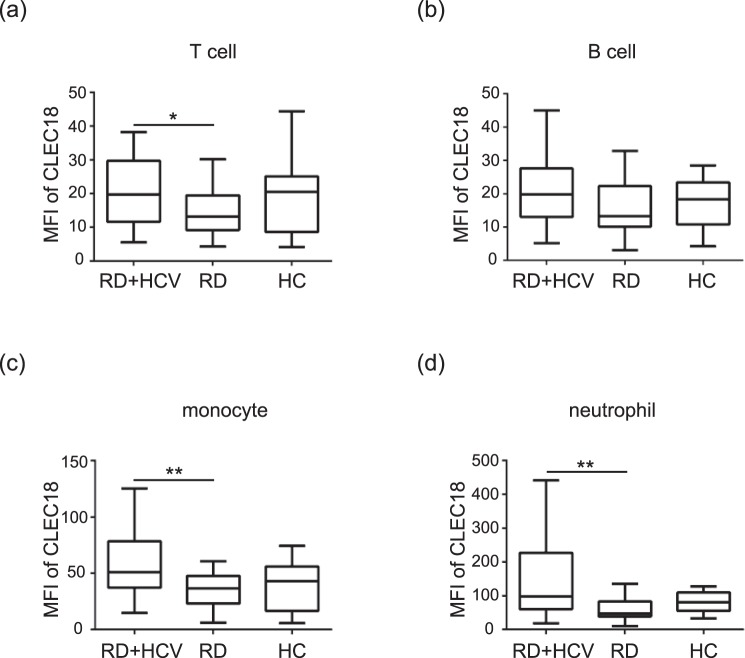
Increased CLEC18 expression levels in T cells, monocytes and neutrophils of rheumatic patients with chronic HCV infection. The intracellular CLEC18 expression levels in (**a**) T cells (CD3), (**b**) B cells (CD19), (**c**) monocytes (CD14), and (**d**) neutrophils (CD66b) were obtained from the peripheral blood of each represented cohort using a flow cytometry assay. RD + HCV, rheumatic patients with HCV infection (n = 41); RD, patients with rheumatic diseases (n = 45); HC, healthy control (n = 14). MFI, mean fluorescence intensity. The Dunn-Bonferroni test was used for between-group comparison of the expression levels of CLEC18. *P < 0.05, **P < 0.01.

We further analyzed the distribution and expression of CLEC18 in different immune cells using flow cytometry. Significantly higher levels of CLEC18 in circulating T cells (CD3^+^), monocytes (CD14^+^), and neutrophils (CD66b^+^) were observed in rheumatic patients with HCV infection (n = 41, median 19.79, interquartile range [IQR] 11.65–29.68; 50.96, IQR 37.19–78.42; 97.57, IQR 60.25–227.06; respectively) compared with those without infection (n = 45, 12.00, IQR 9.15–19.47, *P* < 0.05; 36.54, IQR 23.00–47.81, *P* = 0.001; 46.80, IQR 38.72–82.99, *P* < 0.001; respectively, Fig. 2a,c and d). However, there was no significant difference in CLEC18 levels in circulating B cells (CD19^+^) (Fig. 2b). Additionally, there was no significant difference in CLEC18 levels in circulating immune cells between the rheumatic patients with HCV infection and healthy controls.

### Correlations between intracellular CLEC18 levels in neutrophils and the presence of HCV-associated mixed cryoglobulinemia syndrome

**Figure 3:**
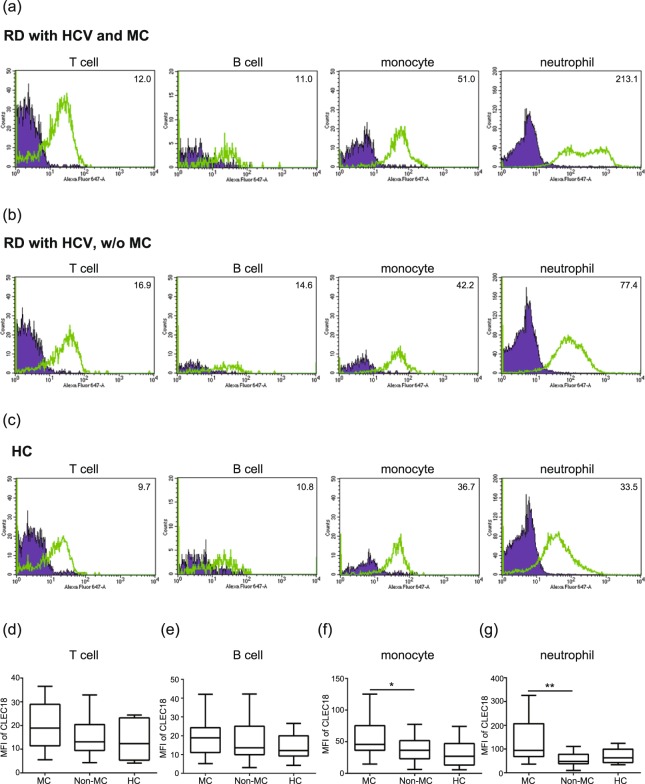
Increased CLEC18 expression in neutrophils of patients with HCV-associated mixed cryoglobulinemia (MC). The intracellular CLEC18 expression levels in T cells (CD3), B cells (CD19), monocytes (CD14), and neutrophils (CD66b) were obtained from the peripheral blood of each represented individual using flow cytometry assay. (**a**) MC, rheumatic patients with HCV infection and HCV-associated MC; (**b**) non-MC, rheumatic patients with HCV infection, but without HCV-associated MC; (**c**) HC, healthy control. CLEC18 expression levels in (**d**) T cells, (**e**) B cells, (**f**) monocytes, and (**g**) neutrophils of rheumatic patients with HCV-associated MC (n = 28), without HCV-associated MC (n = 13), and healthy control (HC, n = 14). MFI, mean fluorescence intensity. The Dunn-Bonferroni test was used for between-group comparison of the expression levels of CLEC18. *P < 0.05, **P < 0.01.

To analyze the association of CLEC18 expression and the presence of HCV-associated extrahepatic manifestations, we compared CLEC18 levels in different immune cells of patients with or without mixed cryoglobulinemia syndrome. As shown in Fig. 3a–c, the representative examples of cytometric histograms of CLEC18 levels in circulating T cells, B cells, monocytes, and neutrophils were obtained from one patient with both HCV and MC, one patient with HCV infection but not associated with MC, and one healthy subject, respectively. Significantly increased levels of CLEC18 were observed in circulating neutrophils from patients with HCV-associated MC (median 94.52, IQR 68.26–206.84) compared to those without MC (48.00, IQR 38.15–77.90, *P* = 0.001, Fig. 3g). Slightly higher CLEC18 levels were observed in circulating immune cells of patients with HCV-associated MC compared with those in healthy control, but there was no statistical significance.

**Figure 4:**
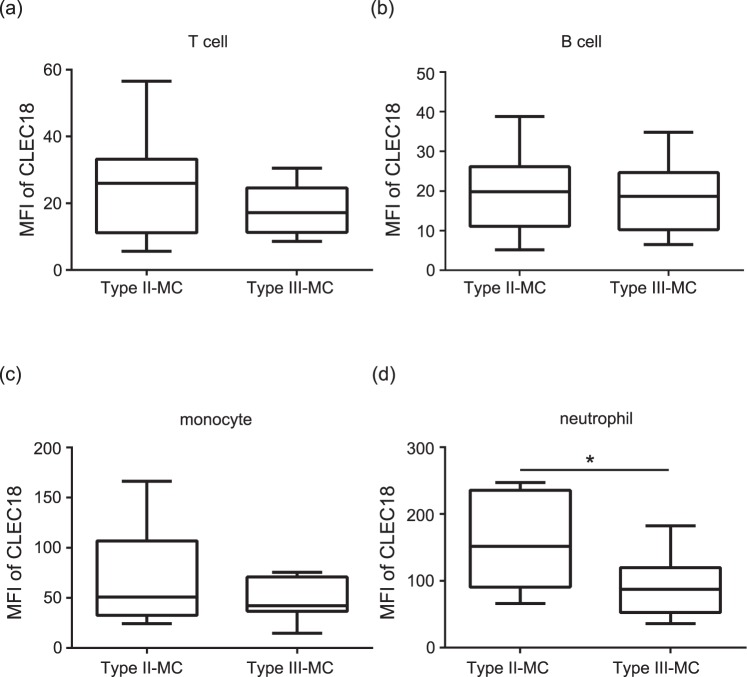
Significantly increased CLEC18 level in neutrophils of patients with type II MC. CLEC18 expression levels in (**a**) T cells, (**b**) B cells, (**c**) monocytes and (**d**) neutrophils of patients with type II (n = 13) or type III (n = 15) mixed cryoglobulinemia (MC). MFI, mean fluorescence intensity. The Mann-Whitney U test was used for between-group comparison of the expressions of CLEC18. *P < 0.05.

Previous studies indicated that type II and III MC, which are associated with chronic virus infection and rheumatic diseases. We further analyzed CLEC18 expression levels in different immune cells from patients with different types of MC. The results showed significantly higher CLEC18 levels in neutrophils from patients with type II MC compared to those with type III MC (median 151.96, IQR 90.66–235.54 vs. 87.09, IQR 52.72–119.63, *P* < 0.05, Fig. 4d). However, there was no significant difference in CLEC18 expression in T cells, B cells, or monocytes between types II and III of MC (Fig. 4a,b and c).

### CLEC18 levels were correlated with cryoglobulin and complement 4 levels

**Figure 5:**
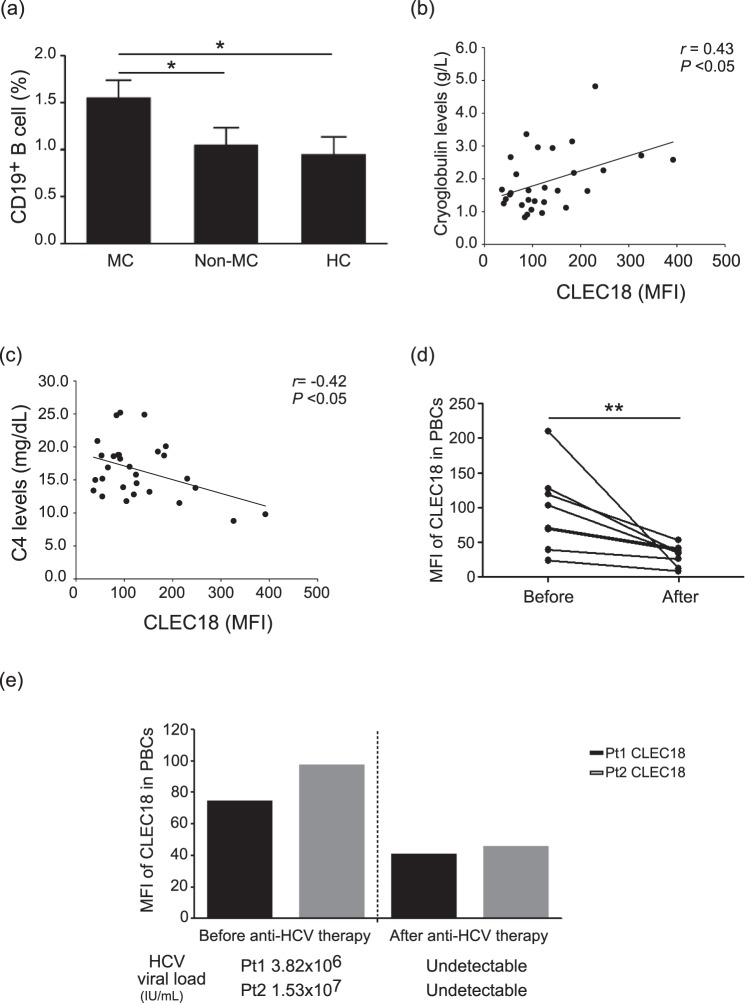
CLEC18 levels were correlated with (**a**) the ratio of CD19^+^ B cells, (**b**) cryoglobulin, and (**c**) complement 4 (C4) levels in rheumatic patients with HCV-associated mixed cryoglobulinemia (MC). Data are presented as the mean ± SEM, rheumatic patients with HCV-associated MC (n = 28) versus rheumatic patients without HCV-associated MC (n = 13) or healthy control (n = 14), determined using the ANOVA test with the Scheffe correction. The correlation coefficient was calculated using Spearman’s correlation test. (**d**) Significant decreases in CLEC18 expression levels in peripheral blood cells (PBCs) (n = 8) paralleled the clinical remission in MC patients after anti-rheumatic therapy. A paired t test was used for between-group comparison of the expression levels of CLEC18. (**e**) The dynamic change of CLEC18 levels in PBCs was positively correlated with the change in HCV RNA levels in serum that paralleled the clinical remission in MC patients after anti-HCV therapy (n = 2). *P < 0.05, **P < 0.01.

We further analyzed the correlation between CLEC18 levels and MC-related factors, including CD19^+^ B cell counts, cryoglobulin levels, and C4 levels. As shown in Fig. 5a, an approximately 1.55-fold increase in CD19^+^ B cell count was measured in patients with HCV-associated MC syndrome compared to those without MC syndrome or HC subjects (*P* < 0.05). Among MC patients, there was a significant positive correlation between CLEC18 expression and cryoglobulin levels (*r* = 0.43, *P* < 0.05, Fig. 5b). Conversely, there was a negative correlation between CLEC18 levels and C4 levels (*r* = −0.42, *P* < 0.05, Fig. 5c).

### Changes in CLEC18 expression levels in patients with HCV-associated mixed cryoglobulinemia after anti-rheumatic or anti-viral therapy

To determine whether CLEC18 expression levels were correlated with disease activity in HCV-associated MC, we examined CLEC18 expression levels in patients with HCV-associated MC before and after anti-rheumatic therapy. Our results showed significantly decreased CLEC18 expression, particularly in neutrophils, in eight patients after anti-rheumatic therapy (before vs. after: 326.0 vs. 44.6 MFI). All patients received conventional synthetic disease-modifying anti-rheumatic drugs (csDMARDs) or corticosteroid treatment (Table 1). Among these patients, two had biologic therapy (one received a TNF inhibitor, etanercept, and the other received rituximab treatment). Additionally, two patients received ombitasvir/paritaprevir/ritonavir (Viekirax^®^), an oral antiviral therapy. The results showed significantly decreased CLEC18 levels in PBCs after anti-rheumatic therapy or upon completion of oral antiviral treatment (Fig. 5d, *P* < 0.01). We also detected a dynamic change of HCV RNA levels in two patients before and after anti-HCV therapy. The results showed a decreased trend of HCV RNA levels in the serum of two patients after therapy (Fig. 5e). Additionally, the dynamic change of CLEC18 levels was positively correlated with the change in HCV RNA levels (Fig. 5e), which was consistent with the results shown in Fig. 1c.

## Discussion

CLEC18 is a novel secretory lectin that is abundantly expressed in hepatocytes and immune cells with differential glycan-binding specificity. In the present study, we first demonstrated significantly higher CLEC18 levels in circulating PBCs from rheumatic patients with chronic HCV infection, particularly in those with cryoglobulinemia syndrome. Our results showed that elevated CLEC18 levels were positively correlated with HCV viral loads in serum. Furthermore, CLEC18 levels were correlated with cryoglobulin and C4 levels. A significant decrease in CLEC18 expression paralleling the reduction of disease activity was observed in patients with HCV-associated MC after anti-rheumatic or antiviral therapy. Based on these observations, we speculate that HCV infection induced CLEC18 expression and may be a potential indicator of HCV infection. Increased CLEC18 expression is associated with increased cryoglobulin levels, which may be related to the occurrence of MC syndrome. Further studies are required for confirmation of these findings.

The innate immune system provides an immediate line of defense against HCV infection, triggering inflammation and playing a critical role in activating adaptive immunity^22^. The function of innate immune cells is closely linked to the recognition of pathogen-associated molecular patterns by immune proteins, which act as PRRs^23^. These PRRs include pentraxins and defense collagens, such as C-type lectins and ficolins^23–25^. C-type lectins play a crucial role in initiating innate immunity and link pathogen recognition to the development of adaptive immunity^26^. Mannan-binding lectin (MBL) belongs to the C-type lectin family, which is a pattern recognition molecule of the innate immune system that binds to the HCV glycosylated envelope proteins, E1 and E2^27^. Brown *et al*. have reported increased MBL levels in HCV patients compared with healthy controls^28^. In the present study, our *in vitro* cell-based results indicated that CLEC18 expression is associated with HCV infection, but the real biological function of CLEC18 in HCV infection has not been thoroughly elucidated. Additional larger or in-depth studies are needed to confirm our conclusions.

Mixed cryoglobulinemia is a common HCV infection-related extrahepatic manifestation. The major characteristic of MC is intravascular deposition of cryoglobulin immune complexes containing IgM rheumatoid factor, polyclonal IgG, and viral RNA^29^. The deposited cryoglobulin can activate the complement system and cause tissue damage and eventual end-organ failure, particularly of the skin and kidneys^30^. Cryoglobulins are single or mixed immunoglobulins that are conventionally classified according to their immunochemical composition, including type I (monoclonal immunoglobulins only), type II mixed cryoglobulins (a mixture of monoclonal and polyclonal immunoglobulins), and type III mixed cryoglobulins (polyclonal immunoglobulins only)^29^. Type I cryoglobulins are frequently associated with hematological disorders^31^. Type II and III mixed cryoglobulins are both associated with chronic virus infection and systemic autoimmune rheumatic diseases^29^. Among these cryoglobulins, type II cryoglobulins are strongly associated with chronic HCV infection^29^. Our results showed significantly increased CLEC18 levels in neutrophils from patients with type II MC compared to those with type III MC (*P* < 0.05), suggesting that CLEC18 expression is associated with HCV infection.

Previous studies demonstrated that the C-type lectin member MBL could bind certain glycoforms of immunoglobulins (Igs), including IgG, IgM, and IgA^32^. Recently, we demonstrated that CLEC18 is a secretory protein and has differential glycan-binding specificity^21^. In the present study, our clinical data showed a significant association between CLEC18 expression and the occurrence of MC syndrome. In addition, a previous study demonstrated that chronic HCV infection is associated with the expansion of the B cell subpopulation and that this expansion is associated with increased production of RF and cryoglobulins^33^. We also observed significantly increased CD19^+^ B cell counts in patients with HCV-related MC compared to those without MC or healthy controls. Further analysis showed a positive correlation between CLEC18 expression and cryoglobulin levels (*r* = 0.43, *P* < 0.05). The evidence above suggest that CLEC18 may be involved in immune complex formation in MC pathogenesis. Further in-depth studies are needed to confirm this possibility.

Complement is central to innate humoral immunity and contributes to clearance of viral infections. In this study, our results showed a negative correlation between CLEC18 and C4 levels. MBL acts as the recognition molecule of the lectin pathway of complement activation by binding to C3 and C4^34^. The levels of C3 and C4 decline following the activation of the lectin pathway. HCV infection–induced complement activation occurs through the lectin pathway, which has been demonstrated to be associated with the pathogenesis of MC^28^. Therefore, patients with cryoglobulinemia often have low levels of complement (hypocomplementemia)^35^, which may explain the significantly lower C4 levels observed in our rheumatic patients with MC compared to those without MC.

Sidharthan *et al*.^30^ demonstrate that PBMCs from patients with HCV-associated MC have an increased interferon-stimulated gene (ISG) expression compared with PBMCs from HCV-infected patients without MC. Their data showed that the gene expression profile of HCV-associated MC reflects compromised neutrophil function, impaired chemotaxis, and an enhanced endogenous interferon gene signature. Given that neutrophils express a large number of cell surface receptors for the recognition of pathogen invasion, including C-type lectins (e.g., CLEC2, CLEC5A)^14,36,37^, chronic stimulation of HCV infection may result in an excessive or inappropriate activation of these PRRs. The PRRs’ inappropriate activation also contributes to the expansion of monoclonal B cells with cryoglobulin production following tissue damage in inflammatory diseases^37^. These findings may support our results, which show elevated levels of CLEC18 in PBCs, particularly in neutrophils from our HCV-infected patients with MC. In addition, increased CLEC18 levels in PBCs corresponded with an elevation of HCV viral loads in our MC patients with HCV reactivation after receiving rituximab therapy, while significantly decreased CLEC18 levels were found in MC patients after anti-rheumatic or antiviral therapy. These observations also support a potential link between CLEC18 and HCV-associated MC. However, the role of CLEC18 in the pathogenesis of HCV-associated MC requires additional confirmatory studies.

To the best of our knowledge, this report describes the first pilot study to investigate the association between CLEC18 expression in HCV infection and the presence of extrahepatic manifestations. Although we have revealed a number of novel findings, this study has some limitations. First, the study included a small number of cases. Therefore, the study is likely not to reflect the complete characteristics of chronic HCV infection in rheumatic patients. Second, this study was cross-sectional in design; thus, we cannot rule out the possibility that CLEC18 expression changed due to the therapeutic strategies. Third, this study lacked HCV infection subjects without rheumatic diseases, and the number of healthy control subjects was small. Therefore, there was no statistical significance of CLEC18 levels in the immune cells from patients with both rheumatic diseases and HCV infection compared with healthy controls. However, we performed *in vitro* cell-based assays to demonstrate that increased CLEC18 expression was detected in HCV-infected cells compared with that in uninfected cells. Future studies focusing on CLEC18 *ex vivo* and an in-depth analysis of the pathogenic mechanisms in HCV infection are needed.

In conclusion, we found that a novel C-type lectin member, CLEC18, is significantly associated with HCV infection in rheumatic patients, particularly in those with HCV-associated MC syndrome. Based on our observations, we speculate that CLEC18 may act as a potential indicator of HCV-associated MC syndrome. Additionally, CLEC18 levels are positively correlated with HCV viral loads, which suggests that CLEC18 may be a novel target for anti-HCV therapeutic purposes. This application requires confirmation from further larger and in-depth studies.

## Methods

### Cells and HCV infection

Huh7.5 cells were cultured in Dulbecco’s modified Eagle’s medium (DMEM) supplemented with 10% fetal bovine serum, nonessential amino acids, 100 units/mL penicillin, and 100 mg/mL streptomycin at 37 °C in a 5% CO_2_ incubator. Huh7.5 cells were infected with the HCV JC1 strain at a multiplicity of infection (MOI) of 5 at 37 °C. At 72 h postinfection, cells were collected and analyzed for intracellular CLEC18 levels by flow cytometry.

### Anti-CLEC18 mAbs

CLEC18 mAbs were obtained as described previously^14^. Briefly, BALB/c mice were immunized with purified recombinant hCLEC18A.Fc fusion protein for the production of mAbs. Selected appropriate mice and isolated lymphocytes from the immunized mouse spleen were fused with mouse myeloma NS-1 cells. Fused cells were cultured in a HAT selection medium, and the medium was changed after 1 week. After 2 weeks, the culture supernatants were collected for screening using ELISA to determine the candidate clones for further analysis by limiting dilution. Anti-CLEC18A mAbs were selected using ELISA-based differential screening, and the clone recognizing recombinant CLEC18A.Fc but not human IgG1 were considered positive clones.

### Study Design

This prospective study was conducted at Taichung Veterans General Hospital, a medical center in Taiwan, from March 2016 to February 2018. The inclusion criteria for patients with rheumatic diseases were as follows: (1) the age for study entry should be at least 20 years; (2) subjects should meet the 2002 revised version of the European criteria for Sjögren’s syndrome (SS)^38^, the 2010 revised criteria of the American College of Rheumatology (ACR) for rheumatoid arthritis (RA)^39^, and the 1997 revised criteria of the ACR for systemic lupus erythematosus (SLE)^40^; (3) and subjects could sign the informed consent after full explanation. All of the subjects underwent a medical history, clinical examination, laboratory standard tests, serological markers of hepatitis B virus, and HCV detection. The exclusion criteria in this study were as follows: (1) patients with positive HBsAg; (2) subjects with a habit of alcohol consumption; (3) subjects with concomitant malignancy; (4) subjects with bacterial infection within one week of enrollment time. Serum alanine aminotransferase (ALT) levels and HCV viral loads were measured before and after anti-rheumatic disease therapy. This study was conducted in compliance with the Declaration of Helsinki and has been approved by the Institutional Review Board of TCVGH (SF16036B). The methods were carried out in accordance with the approved guidelines, and written consent from all the participants was obtained.

### Serological and virological evaluation for HCV infection

HCV infection was diagnosed by using commercial 3^rd^ generation ELISA kits (J. Mitra & Co., Pvt. Ltd., New Delhi, India) to detect anti-HCV antibodies, and subsequent measurement of HCV viremia by polymerase chain reaction if anti-HCV antibodies were detected. Serum HCV viral loads were quantified by using the Roche Cobas TaqMan HCV Test (Roche Diagnostics, Switzerland) according to the manufacturer’s instructions. Viral load was expressed as log10 of the detected values for analysis.

### Determination of serum cryoglobulinemia

Diagnosis of cryoglobulinemia was defined by the presence of cryoglobulins in serum stored at 4 °C for 10 days in two fractions, and reversibility of the cryoprecipitation in one fraction replaced at 37 °C when a cryoprecipitate is formed^29,41^. The classification of serum cryoglobulinemia was determined by immuoelectrophoresis^29^.

### Flow cytometry analysis for CLEC18 levels

For flow cytometry analysis, intracellular staining for CLEC18 was performed following fixation and permeabilization with IntraPrep Permeabilization Reagent (Beckman Coulter, Brea, CA, USA) using the modified method of a previous study^21^. Cells were incubated with the Alexa Fluor 647–conjugated anti-CLEC18 monoclonal antibody (clone 3A9E6), and examined by flow cytometer (FACSCanto II, BD Biosciences). Alexa Fluor 647–conjugated IgG1 (R&D Systems, Minneapolis, USA) was used as an isotype control. Data were analyzed by the CellQuest software and were expressed as the mean fluorescence intensity (MFI) of CLEC18.

### Measurement of serum CLEC18 levels

CLEC18 levels in serum were quantified using ELISA according to the standard operating procedure. Briefly, the CLEC18 antibody (clone 3F10F8) was coated onto 96-well microplates (50 μl/well, Corning) and incubated for 16–18 h at 4 °C, and then incubated with 200 μl of blocking buffer (Tris-buffered saline [TBS] with 1% polyvinyl alcohol) for 1 h at room temperature before washing twice with TBST (TBS with 0.05% Tween 20). Each well was incubated with 50 μl of the patient’s serum or standard for 2 h at room temperature before washing with TBST three times. After washing, the well was incubated with 50 μl of biotin-conjugated CLEC18 monoclonal antibody (clone 3A9E6) for 2 h at room temperature before washing with TBST three times. Finally, each well was incubated with 50 μl of streptavidin-HRP for RT 20 min, followed by the addition of 100 μl of tetramethylbenzidine substrate (BD Biosciences) for 15 min; the reaction was stopped by addition of 1 N sulfuric acid prior to analysis using an ELISA reader. All of the samples and standards were measured in duplicate, and concentrations were determined from a standard curve using mean optical density values. Serum CLEC18 levels were expressed as ng/mL.

### Statistical analysis

Results are presented as the mean ± standard deviation (SD) or standard error of mean (SEM). The analysis of variance (ANOVA) test, Dunn-Bonferroni test, Mann-Whitney U test and paired t test were used for between-group comparisons of the expression of CLEC18. The ANOVA test was used to verify the association of CLEC18 and HCV infection or HCV-associated MC. Dunn–Bonferroni test was used to analyze the distribution and expression of CLEC18 in different immune cells from patients with different diseases (rheumatic patients with or without HCV infection and healthy subject) or different HCV-associated MC syndromes (MC, Non-MC and healthy subject). Mann–Whitney U test was used to analyze the expression of CLEC18 in different immune cells from MC patients with different MC typing. A paired t test was used for comparison of CLEC18 expression between patients before and after therapy. The correlation coefficient was calculated using Spearman’s correlation test. *P* values < 0.05 were considered to be statistically significant.
